# Imaging in Non-Traumatic Emergencies

**DOI:** 10.3390/tomography9030093

**Published:** 2023-06-12

**Authors:** Mariano Scaglione, Salvatore Masala, Francesca Iacobellis, Michele Tonerini, Giacomo Sica, Carlo Liguori, Luca Saba, Stefania Tamburrini

**Affiliations:** 1 Radiology Department of Surgery, Medicine and Pharmacy, University of Sassari, Viale S. Pietro, 07100 Sassari, Italy; 2 Department of Radiology, James Cook University Hospital & Teesside University, Marton Road Marton Rd., Middlesbrough TS4 3BW, UK; 3 Department of General and Emergency Radiology, “A. Cardarelli” Hospital, Via Cardarelli 9, 80100 Napoli, Italy; 4 Department of Emergency Radiology, Cisanello Hospital, Via Cisanello, 56124 Pisa, Italy; 5 Department of Radiology, Monaldi Hospital, 80131 Naples, Italy; 6 Ospedale del Mare-ASL NA1 Centro, Via Enrico Russo, 11, 80147 Napoli, Italy; 7 Department of Medical Sciences, University of Cagliari, 09124 Cagliari, Italy

## 1. Introduction

“Emergency” is a scenario that every medical professional must face since the first day of her/his career. The term “emergency” refers to a serious, often progressive clinical situation that calls for immediate diagnostic and therapeutic action. Although this is an old issue, approaches to this critical issue have deeply changed in the last three decades thanks to the impact of modern imaging, which has revolutionized the clinical “classic paradigm” based on observation, physical examination and diagnosis [1,2,3,4,5,6,7,8,9,10]. Today, diagnosis via imaging has almost entirely replaced physical examination in the emergency room, and the Radiologist has become of primary importance in this setting. However, close co-operation among the various specialists involved is essential for successful patient management, and thus the Radiologist needs to have a full understanding of all the imaging modalities and technical skills required, as well as appropriate clinical knowledge of the disorder in order to manage the condition [9,11,12,13,14,15,16]. This Special Issue aims to provide a review of the multifaceted etiology, pathophysiology and clinical presentation of some emergency conditions in different scenarios, focusing on the imaging features that are relevant to a timely management approach.

## 2. The Special Issue at a Glance

This Special Issue—“Imaging in non-traumatic emergencies”—consist of nine state-of-the-art articles by well-known experts in the field of emergency radiology. In the article “Contrast enhanced ultrasound compared with MRI and CT in the evaluation of post-renal transplant complications”, E. David et al. [17] illustrate the possibilities of contrast-enhanced ultrasound (CEUS) in the early detection of post-renal transplant complications. This imaging modality is a relatively new approach compared to the classic imaging modalities such as MR and CT, which have a higher cost and lower accessibility. There are also possible negative impacts on the patient’s health, including nephrogenic systemic fibrosis and contrast-induced nephropathy (CIN) caused by gadolinium and iodine contrast agent, respectively, as well as issues with radiation dose associated with the use of CT. Therefore, considering the overall advantages and disadvantages, CEUS can currently be considered an effective first-line imaging modality for post-operative early and long-term follow-up in renal transplant, thus reducing the need for biopsies, and providing adequate guidance for drainage procedures.

The article entitled “multidetector computed tomography (MDCT) findings of complications of acute cholecystitis. A pictorial essay” by F. Sandomenico et al. [18] gives an overview of one of the most common surgical pathologies that every radiologist should consider in the differential diagnosis of right upper quadrant pain. However, although US still represents the first imaging modality, it sometimes struggles to find serious complications that contrast-enhanced MDCT does not miss, due to its higher sensitivity, specificity and capabilities of the post-processing techniques.

“Spontaneous Retroperitoneal Hematoma Treated with Percutaneous Transarterial Embolization in COVID-19 Era: Diagnostic Findings and Procedural Outcome”, by F. Tiralongo et al. [19], aims to evaluate spontaneous retroperitoneal hematomas in the COVID-19 era, focusing on the imaging features of CT-angiography and Digital Substraction Angiography, and on the safety, as well as technical and clinical success, of Trans Arterial embolization (TAE), comparing patients affected by COVID-19 and non-COVID-19 patients. The study demonstrates that TAE should be considered an important, safe, effective and potentially life-saving option for the management and the treatment of patients affected by COVID-19 who present with spontaneous retroperitoneal hematoma and who would not benefit from conservative treatment.

The article “Role of CT and MRI in cardiac emergencies” by C. Liguori et al. [20] focuses on the impact of CT and MRI on diagnostic workflow of acute chest pain, demonstrating the reduction rate of possible fatal consequences of a missed diagnosis, malpractice costs of missed acute coronary syndromes and unnecessary hospital admissions every year. CT provides consistent diagnostic support, mainly for suspected coronary disease in patients with a low or intermediate pre-test risk. Moreover, it can obtain information in the case of cardiac involvement in pulmonary vascular obstructive disease. MRI, on the other hand, has a leading role in the condition of myocardial damage, irrespective of the underlying inflammatory or stress-related etiology.

The article entitled “Systemic Emergencies in COVID-19 Patient: A Pictorial Review” by M. Albanesi et al. [21] provides a systematic approach based on an imaging review of all major multi-organ manifestations of COVID-19 infection, emphasizing the role of the radiologist in an emergency setting in providing both a safe and accurate diagnosis and a timely tailored management approach.

“The “Black Pattern”, a Simplified Ultrasound Approach to Non-Traumatic Abdominal Emergencies”, by S. Tamburrini at al. [6], suggests a new approach to the search for fluid, which is usually anechoic and thus appears “black” during US, in non-traumatic patients for point-of-care US (POCUS) on the basis of three step analysis: 1. Look for black areas where they should not be. This means searching for effusions or collections. 2. Check if the black areas are too large. This means evaluating anatomical landmarks where fluid should normally be present but may be abnormally abundant. 3. Look for black areas that are not clearly black [19]. This means evaluating fluid aspects, whether wholly anechoic or not (suggesting heterogeneous or corpusculated fluid) [7]. “The black pattern approach” appears to be very useful, and not only in the context of non-traumatic emergencies. It can be applied to a broad spectrum of clinical abnormalities, and thus represents a valuable tool for radiologists, emergency physicians, sonographers and every medical professional involved in a variety of non-traumatic emergency scenarios.

CT pulmonary angiography (CTPA) is an imaging technique that has come to be used daily in cases of suspected acute pulmonary embolism (PE) in emergency departments. Several studies have been conducted on the predictive value of CTPA on the outcomes of PE. The article “Acute Pulmonary Embolism: Prognostic Role of Computed Tomography Pulmonary Angiography” by G. Zantonelli et al. [22] provides an updated review of the literature, and reports imaging parameters and quantitative CT scores to predict the severity of PE.

“MDCT Imaging of Non-Traumatic Thoracic Aortic Emergencies and Its Impact on Diagnosis and Management—A Reappraisal” by T. Valente et al. [23] deals with the tricky topic of non-traumatic thoracic aorta emergencies which have been grouped under the common term “acute aortic syndromes”. Aortic diseases, which include aortic dissection and its variants, are life-threatening conditions clinically indistinguishable on presentation. Patients with aortic dissection may present with a wide variety of symptoms secondary to the pattern of dissection and end organ malperfusion. Other conditions may be seen in patients with acute symptoms, including ruptured and unstable thoracic aortic aneurysm, iatrogenic or infective pseudoaneurysms, aortic fistula, acute aortic thrombus/occlusive disease and vasculitis. Imaging plays a pivotal role in the patient’s management and care. While chest X-ray remains the initial imaging test in the red room, offering a screening evaluation for alternative common differential diagnoses, state-of-the-art multidetector computed tomography angiography (MDCT-a) is a widely available, rapid, replicable, noninvasive form of diagnostic imaging, with sensitivity approaching 100%. MDCT-a is an impressive tool in the decision-making process with a deep impact on treatment, including endovascular or open surgical or conservative treatment. This article will help every radiologist become familiar with the wide spectrum of these entities to help triage patients appropriately and efficiently.

“Intracranial Hemorrhage from Dural Arteriovenous Fistulas: What Can We Find with CT Angiography?” by A. Negro et al. [24] discusses a relatively rare acquired intracranial vascular malformation that presents with a variety of clinical signs and symptoms, which sometimes makes diagnosis very difficult. In this paper, the authors’ goal was to verify the accuracy and utility of contrast-enhanced brain CT angiography (CTA) for the identification and characterization of dural arteriovenous fistulas (DAVFs) in patients who presented with brain hemorrhage compared to 3D digital subtraction angiography (3D DSA) and MRI. CTA proved to be a valid alternative diagnostic method to 3D DSA for the study of DAVF in the initial and preliminary diagnostic approach, especially in the emergency setting. The use of CTA should be strongly recommended, as it represents a fast, cheap, non-invasive, and above all, easily accessible and available diagnostic technique, unlike DSA or MRI. This allows it to provide all information necessary for the identification, classification and treatment planning of DAVFs.

## 3. Closing Remarks

“Emergencies” will always have multifaceted implications, ranging from patient’s health and safety to costs [25]. Imaging has deeply changed the approach to emergencies, becoming a crucial tool to reach a timely diagnosis and a tailored management. For this reason, the radiologist represents a 24/7/365 member of the emergency team, becoming known as “the doctor’s doctor” because the vast majority of critical decisions depends on his/her decisions. However, the radiologist must be aware of all the possibilities that imaging technology developments can offer every day, including artificial intelligence and deep learning [26].
